# A comparative analysis of the impact of different treatments on Luffa Cylindrica fiber tensile properties

**DOI:** 10.1371/journal.pone.0325237

**Published:** 2025-07-18

**Authors:** Rania Saadeh, Ayman A. Dawod, Ibrahim Mahariq, Ahmad Qazza, Abdelkader Khentout, Mohamed Kezzar, Mohamed Rafik Sari

**Affiliations:** 1 Department of Mathematics, Faculty of Science, Zarqa University, Zarqa, Jordan; 2 Faculty of Information Technology, Zarqa University, Zarqa, Jordan; 3 Department of Mathematics, Saveetha School of Engineering, Saveetha Institute of Medical and Technical Sciences, Saveetha University, Chennai, Tamil Nadu, India; 4 Najjad Zeenni Faculty of Engineering, Al Quds University, Jerusalem, Palestine; 5 University College, Korea University, Seoul, South Korea; 6 Department of Medical Research, China Medical University Hospital, China Medical University, Taichung, Taiwan; 7 Drilling and MCP Department, University Kasdi Merbah, Ouargla, Algeria; 8 Materials and Energy Engineering Laboratory (LMGE), Technology Department, Faculty of Technology, 20 Aout 1955 University of Skikda, Skikda, Algeria; 9 Mechanics of Materials and Plant Maintenance Research Laboratory (LR3MI), Mechanical Engineering Department, Faculty of Engineering, Badji Mokhtar University of Annaba (UBMA), Annaba, Algeria; King Mongkut's University of Technology North Bangkok, THAILAND

## Abstract

In recent years, there has been a growing demand for environmentally friendly materials as alternatives to synthetic fibers, which pose significant environmental hazards due to their non-biodegradability. Natural fibers, such as *Luffa Cylindrica*, have gained attention due to their sustainability, lightweight properties, and biodegradability. However, their poor mechanical performance in composite applications limits their widespread use. The novelty of this study lies in the exploration of various treatments aimed at improving the tensile properties of *Luffa Cylindrica* fibers (*LCFs*), a relatively under-explored natural fiber. The research investigates the effects of immersion treatments in mineral water, seawater, vinegar (CH₃COOH), sodium bicarbonate (NaHCO₃), and ethanol (C₂H₆O) on the tensile strength, surface morphology, and absorption properties of these fibers. The study highlights that untreated *LCFs* exhibit superior tensile strength (5.59 MPa) compared to treated fibers. Additionally, the statistical analysis utilizing two-parameter Weibull distribution offers novel insights into the mechanical properties of treated *LCFs*, confirming significant variations in fiber behavior with different treatments. This study contributes to the understanding of surface modifications that enhance the performance of *Luffa Cylindrica* fibers in composite materials, a promising step towards their industrial application.

## 1. Introduction

Environmental awareness in the modern world has made it easier for scholars and researchers to study and create new materials. It has also prompted scientists to investigate novel natural fibers as a substitute for synthetic ones. When disposed of, synthetic fibers such as Kevlar, nylon, glass fibers, etc., provide serious environmental hazards [1–3]. It is a non-biodegradable substance that affects the quality of the air and water and adds to landfills [4]. Therefore, scientists have been compelled by the demand for environmentally benign materials to create biocomposite materials for a variety of industries, including healthcare, aviation, construction, and automotive sectors, windows, doors, insulating panels, thermal insulators, aircraft, etc. [5–7]. Since natural fiber reinforced composites are lightweight, nontoxic, inexpensive, and biodegradable, they are favored over synthetic fiber composites [8]. In recent years, natural fibers have become more and more popular as an economical and ecologically beneficial substitute for synthetic fibers when it comes to reinforcing composite products [9–11]. Natural fibers are abundant, lightweight, biodegradable, environmentally friendly, inexpensive, and have a high specific stiffness, among other desirable qualities [12]. Other possible uses for natural fibers include acoustic insulators in recording studios and dampers to stop vibration. Because environmentally friendly materials are needed, efforts are being made to find new resources and investigate their possible uses [13].

Overcoming some constraints, such as hydrophobicity and fiber wettability, is necessary to develop better and more sustainable composites [14]. Surface modification methods including NaOH treatment, silane, potassium permanganate, benzoyl chloride, plasma therapy, laser treatment, corona treatment, etc., have been documented in a number of investigations, highlighted possible changes to the fiber surface to enhance its characteristics and interact with the matrix [1,15,16].

The primary components of plant fibers include cellulose, lignin, hemicelluloses, and wax. The chemical composition, tensile strength, thermal stability, quality, and percentage of fiber yield of natural fibers are all influenced by the plant’s location and environmental conditions. The plant’s maturity, the extraction method (mechanical, chemical, or water retting), and the portion of the plant from which the fiber was taken are other variables that affect the fiber’s characteristics [17]. However, natural fibers are also very hydrophilic and absorb a lot of moisture. Prior to the creation of the composite, the hydrophilic character of the fiber must be increased by appropriate surface treatments to strengthen the interfacial bonding strength between the fiber and matrices [18]. Common methods for changing the surface characteristics include gamma irradiation, sea water treatment, silane treatment, acetylation with etherification, alkalization, benzoyl peroxide treatment, potassium permanganate treatment, stearic acid treatment, isocyanate treatment, and acrylation [19]. These procedures can eliminate surface contaminants and activate hydroxyl groups in the natural fiber. The existing production level is unable to fulfill the current demand because of the growing market demand for plant fibers with suitable qualities. This means that in order to meet consumer demand, cellulosic fibers need to be thoroughly investigated to see if they can be employed as a polymer matrix reinforcement. Among these fibers (*Luffa cylindrica* fibers).

Since luffa is a sub-tropical plant, it needs moderate summer temperatures and a long growing season free of frost in temperate climates [9,20]. In theory, employing natural fibers like Luffa in composites has several benefits, including low density, high flexural strength, flexibility, and high elastic modulus [21]. Mohanta and Acharya [22]. investigated the impact of the fiber surface at room temperature with 5% NaOH, 0.05% potassium permanganate, and benzoyl chloride. It was found that the fibers that underwent chemical treatment enhanced the Luffa-reinforced epoxy composites’ ultimate mechanical characteristics. Chen et al. [23]. examined how the characteristics of high-density Luffa fiber bundles were affected by three softening procedures based on alkali. They found that the fibers’ compressive strength and plateau stress were considerably reduced by the 5% NaOH/5% H2O2 treatment. Nonetheless, the 10% NaOH/20% CH3COOH treatment was thought to be the most effective. Zhang et al. [24]. examined the effects of alkali-urea, alkali-acetic acid, and alkali-hydrogen peroxide on Luffa. It was shown that the treatments significantly increased the fibers’ compressive strength. Premalatha et al. [25]. investigated how to lessen the hydrophobic properties of Luffa fibers by using stearic acid, potassium permanganate, benzoyl peroxide, and NaOH. It was observed that the crystallinity and thermal stability of all modified Luffa fibers improved. However, the thermal stability property was better with the stearic acid treatment. Dhilip et al. [26] examined Chemical treatment can improve the qualities of natural fibers; banana fibers were used unaltered, areca fibers were modified with silane using ultrasonic means, and areca fibers were employed in a variety of stacking configurations. Using a hand layup technique, the tri-layer epoxy composites were created in accordance with four stacking sequences. The created composites’ morphological, mechanical, and flammability properties were examined. The results of Fourier transform infrared spectroscopy show that fibers have changed following silane treatment. A 15% increase in ultimate tensile strength, 20% in hardness, 34% in ultimate flexural strength, and 18% in impact characteristics were the results of morphological studies conducted using a scanning electron microscope, which demonstrated an excellent interfacial bond between the chemically treated fibers and the matrix. This illustrates how surface change affects areca fibers and stacking order. The findings demonstrated that fiber-matrix interaction was essential in regulating the produced composites’ performance attributes.

In the context of numerical methods and theoretical analyses applied in this research, recent studies have contributed significantly to enhancing the understanding of numerical radius inequalities, perturbation of quadrature rules, and fractional derivative modeling, all of which play a critical role in accurately assessing the mechanical properties of natural fibers. Hazaymeh et al. [27] presented refined numerical radius inequalities, which have implications for evaluating errors in computational methodologies related to materials’ mechanical testing and statistical modeling of natural fibers. Their work offers valuable theoretical foundations for numerical evaluations conducted in this study. Additionally, Hazaymeh et al. [28] proposed an innovative perturbed Milne’s quadrature rule, providing essential Lp-error estimates that significantly improve the numerical accuracy of integrations involving differentiable functions, directly relevant to analyzing experimental data such as that presented in our tensile tests.

Furthermore, the analysis of mechanical properties and behavior under fractional derivatives, as explored by Alzahrani et al. [29], highlights advanced numerical techniques that could offer insights into complex dynamic systems and chaotic behaviors, which might be analogously applied to the intricate structure and behavior of fibers under various treatment conditions. Additionally, foundational theoretical insights into the existence and stability of solutions for differential and integral equations provided by Qazza and Hatamleh [30] and Qazza et al. [31] respectively, underpin the statistical and mechanical reliability assessments conducted in this study, emphasizing the importance of robust mathematical modeling in interpreting the results of natural fiber characterization.

These recent contributions not only reinforce the theoretical basis of the current research but also indicate promising directions for further investigation into advanced numerical methods and mathematical frameworks applicable to natural fiber composites and related fields.

Although various surface treatment methods have been explored for improving the properties of natural fibers, *Luffa Cylindrica* fibers (*LCFs*) have not been extensively studied to the effects of different treatments on their tensile properties and overall performance in composite materials. Previous studies have focused on individual treatment methods such as alkali treatment or chemical modifications, but there remains a lack of comprehensive research on the combined effect of multiple treatments, especially those involving natural and environmentally friendly solutions like seawater, vinegar, and sodium bicarbonate. Furthermore, the influence of these treatments on the fiber’s moisture absorption and surface morphology has not been fully explored.

This study aims to fill this gap by investigating the effects of five different immersion treatments (mineral water, seawater, vinegar, sodium bicarbonate, and ethanol) on the tensile properties, surface morphology, and absorption behavior of *Luffa Cylindrica* fibers. The overall objective is to determine the optimal treatment for enhancing the mechanical performance and surface characteristics of *LCFs*, providing valuable insights for their potential use in eco-friendly composite materials.

This study specifically focuses on the tensile behavior of treated versus untreated *LCFs*, as tensile strength is a critical factor in determining the suitability of fibers for reinforcing composite materials. By enhancing the tensile properties of these fibers through surface treatments, it may be possible to improve their performance in composites, making them more competitive with synthetic fibers in various industries, such as automotive, construction, and packaging. Moreover, understanding the impact of treatments on the fiber’s moisture absorption and surface characteristics can provide insights into how to optimize *LCFs* for use in composite materials that are exposed to varying environmental conditions.

## 2. Materials and methods

### 2.1. Raw materials

Collo, Algeria was the source of the *Luffa Cylindrica* fibers (*LCFs*). The already-dried pods were stripped of their outer bark, revealing a clean fiber that could be studied. used in the study were seawater, vinegar (CH3COOH), sodium bicarbonate (NaHCO3), ethanol (C2H6O), and mineral water. After carefully removing the seeds from the dry fiber framework, the fibers were left to soak in the distilled water at room temperature (25°C) for a full day. After then, the fibers were taken out and allowed to sundry for two days before being subjected to additional testing and characterization.

### 2.2. Composition of *Luffa cylindrica* fiber

The chemical Composition of *Luffa Cylindrica* fibers (*LCFs*)determines how well they work when incorporated into another material. Similar to other biomass, cellulose, hemicellulose, and lignin make up the majority of *Luffa cylindrica* fibers [32]. The cellulose content of *LCFs* was determined using the method described by Kushner and Hoffer [33]. Hemicelluloses were assessed in accordance with ASTM E1755-61 criteria [34]. and the ASTM D1106 standard was followed to assess the two fibers’ lignin content gravimetrically [35]. The cellulose, hemicellulose, and lignin contents were calculated in this research using the following equations (by Wetaka et al. [36]):


$$Cellulose\;content\%=\frac{M_{2}}{M_{1}}\times 100$$ (1)

$$Hemicellulose\%=\frac{W_{2}}{W_{1}}\times 100$$ (2)

$$Lignin\%=\frac{M_{i}}{M_{f}}\times 100$$ (3)

### 2.3. Morphology analysis

Using a scanning electron microscope (SEM), the longitudinal image of both treated and untreated *Luffa Cylindrica* fibers (*LCFs*) was analyzed to investigate the morphological properties, The voltage range for the scanning electron microscope (HitachiS-4800 microscope, Tokyo, Japan) [37], was 0.3 to 30 kV. The untreated and treated fibers were coated with an ion sputter coater with a gold target to acquire images of good quality [38], and finally, the resulting images were captured and recorded in the magnification of 50 μm.

### 2.4. Absorption test

The purpose of this *Luffa Cylindrica* fibers (*LCFs*) study is to examine how the mass of the sample varies over time when it is submerged in C2H6O, CH3COOH, NaHCO3, mineral water, and seawater at room temperature (25°C). Over the course of 21 days, or 504 hours (adapting the immersion technique) [39], the fibers were completely submerged in the water samples, and their mass was periodically assessed. Experiments on water absorption were carried out in compliance with ASTM D570 [40]. This method involves immersing the fibers in various solutions and monitoring the change in mass over a set period of time. The fibers were first dried to a constant weight before immersion and then periodically weighed at predetermined intervals to assess the absorption rate. Weighing was done using an electronic scale with a 0.0001 g precision. Using Equation 4, the weight change of a specimen (*LCFs*) at time (t) during the course of the curing period was computed as a percentage, Mt (%) [3,41,42].


$$M_{t}(\mathrm{\backslash nonumber}\;\%)=\frac{m_{t}-m_{i}}{m_{i}}\times 100$$ (4)

where *m*_*i*_ is the initial weight of the dried samples and *m*_*t*_ is the weight of the fibers at time t.

The results were analyzed to determine the moisture absorption capacity of the fibers, which is crucial for understanding how the fibers interact with various matrices in composite materials. The absorption behavior is influenced by the surface morphology, chemical composition, and treatment method, and it plays a significant role in the overall performance of the fibers in composite applications.

### 2.5. Tensile tests

Before testing, the cutting of the fiber network was performed along the longitudinal length of the Luffa Cylindrica bunch for tensile characterization, four dry samples were taken, 20 samples were taken, divided into five groups, then immersed in mineral water, seawater, CH3COOH, NaHCO3, and C2H6O l for 24 hours, then extracted and dried with absorbent paper. Tensile testing was done using the Test112 universal testing machine. As per Tensile testing was done by ASTM D3379 [43], utilizing a 5 KN speed and a 1 mm/min tangential displacement velocity, the tensile tests were carried out at room temperature. On cardboard end tabs, the single fiber was adhered using a quicksetting polyester glue. The samples were mounted so that each one measured 20 mm in length [44]. Stress-strain curves were produced for each sample after tensile loads were applied until the sample failed. For every test, four specimens were utilized, and the average result is shown in the final results. The main fiber tensile characteristics, both when the fibers were dry and when they were treated differently, were also found.

### 2.6. Statistical analysis

Examination the dispersion of data, an intrinsic feature of *Luffa Cylindrica* fibers (*LCFs*), makes it challenging to assess experimental results from studies conducted on these fibers. The distribution of flaws in the fiber or on the fiber’s surface can be used to explain this variability [45], and so, statistical methods are required to assess its average mechanical characteristics. Using the Minitab 21 software and the two- and three-parameter Weibull model, which was applied by multiple authors for various cellulosic fibers, the experimental data from the uniaxial tensile tests conducted for the *Luffa Cylindrica* fibers (*LCFs*) treated and presented in this work was statistically analyzed. The data was divided into six groups, each of which contained four fiber samples [46]. to calculate their mechanical characteristics.

The following defines the three-parameter Weibull distribution’s cumulative distribution function:


$$F\left(x\right)=1-Exp\left[-{(\frac{x-s_{0}}{s})}^{m}\right]$$ (5)

where m is the shape parameter or Weibull modulus, s > 0 corresponds to the scale parameter (characteristic value), s_0_ is the threshold that reflects an average value of the parameter x, and x, s, s_0_, and m are all positive real. To get meaningful results, stress, Young’s modulus, and elongation at break were measured at a 95% confidence level. Assuming that the threshold is equal to zero (s_0_ = 0), the two-parameter Weibull model is produced.

The tensile property data of films were evaluated using the variance analysis (ANOVA). At a 95% confidence level, the difference between the samples was deemed significant at alpha 0.05.

## 3. Results and discussion

### 3.1. Chemical composition of the *Luffa Cylindrica* fibers

**Fig 1:**
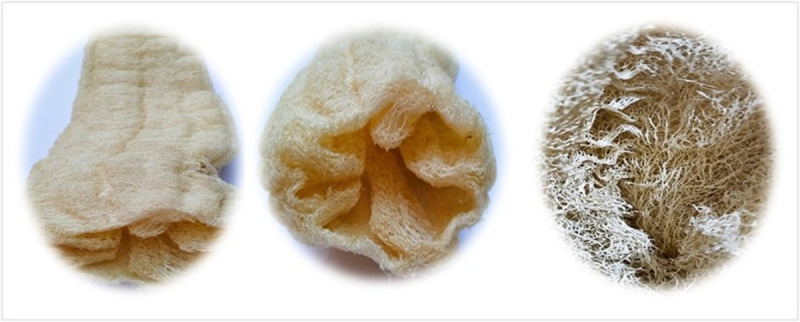
*Luffa Cylindrica* samples.

**Fig 2:**
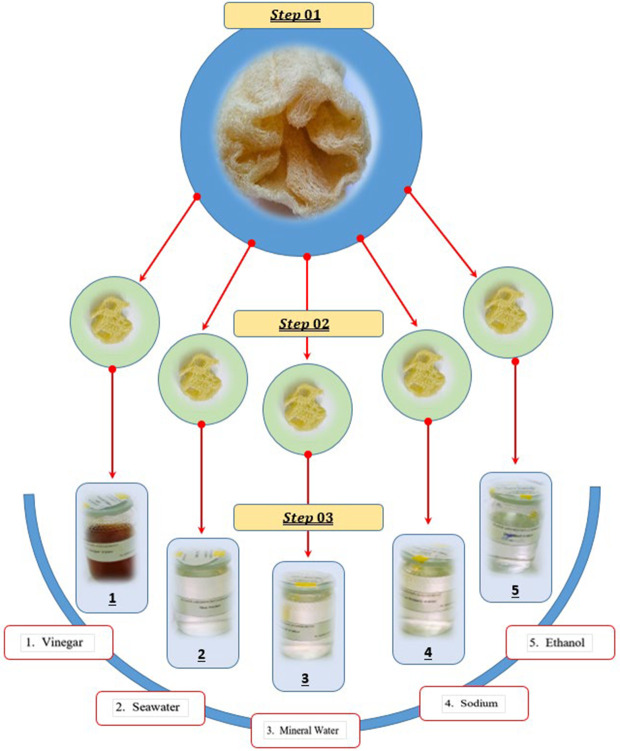
Solutions in which *Luffa Cylindrica* fibers (*LCFs*) have been treated and immersed.

The results of the *Luffa Cylindrica* fibers (*LCFs*) chemical composition are displayed in Table 1. It is evident that the fibers have a higher percentage of cellulose and hemicellulose, suggesting that they have the potential to be used as textile fibers. The findings are consistent with earlier research on *luffa cylindrica* fibers (Table 1) (Figs 1 and 2).

**Table 1:** Chemical composition of *Luffa Cylindrica* fibers (*LCFs*) and comparison with other natural fibers.

Fiber	Cellulose (%)	Hemicellulose (%)	Lignin (%)	References
**Dry fibers**	66.78	19.43	13.51	This study
***LCFs* immersion in mineral water**	66.56	18.79	13.34	
***LCFs* immersion in seawater**	65.89	19.02	13.19	
***LCFs* immersion in CH3COOH**	65.46	18.39	13.33	
***LCFs* immersion in NaHCO3**	65.78	18.65	13.22	
***LCFs* immersion in C2H6O**	65.77	18.49	13.45	
**Jute**	61–71.5	17.9–22.4	11.8–13	[61]
**Hemp**	70.2–74.4	17.9–22.4	3.7–5.7	[62]
**Sisal**	78	10	8	[61]
**Kenaf**	45–57	8–13	21.5	[62]
**Coir**	37	–	42	[62]
**Banana**	63–67.6	19	5	[63]
**Ramie**	68.6–91	5–16.7	0.6–0.7	[64]
**Agave**	68.42	4.85	4.85	[65]
**Sponge gourd outer skin**	81.69	–	10.14	[66]

### 3.2. Morphological characteristics

**Fig 3:**
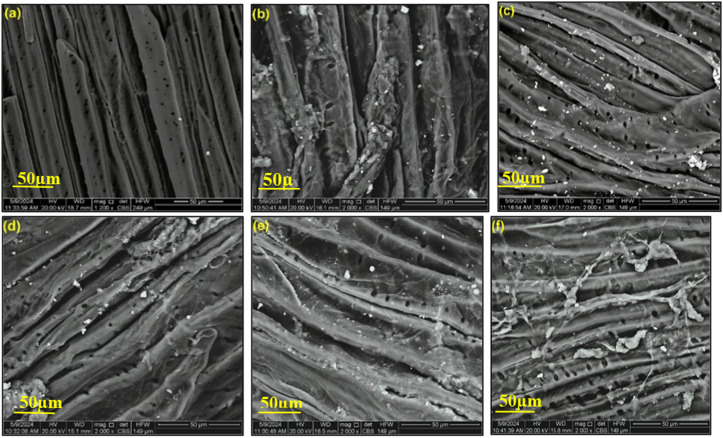
Scanning electron microscopy (SEM) images of the longitudinal position of *Luffa Cylindrica* fibers (*LCFs*): (a) dry fibers, (b) fibers immersion in mineral water, (c) fibers immersion in seawater, (d) fibers immersion in CH3COOH, (e) fibers immersion in NaHCO3, and (f) fibers immersion in C2H6O.

The surface morphology of dry and treated *Luffa Cylindrica* fibers (*LCFs*) is depicted in Fig 3 using SEM characterization pictures. As can be seen in Fig 3a, the surface of the dried fibers contains pores and a smooth surface, while the surfaces of the treated fibers in Fig 3b–f, show that the diameter of the pores is somewhat larger compared to the dry fibers, and the fracture disintegration of the fibers also appears [47]. These pores and fractures, which have different sizes and chapes, may lead to a weakening of the fiber structure and thus a decrease in tensile strength [44,48].

### 3.3. Absorption test analysis

**Fig 4:**
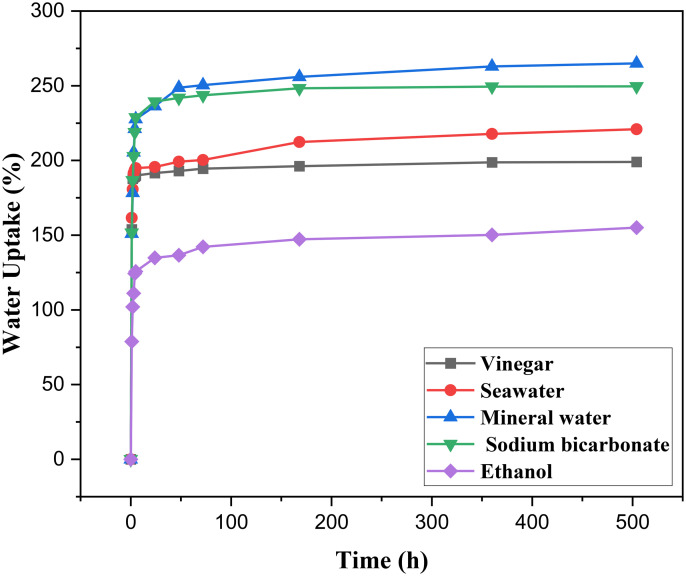
Graphical representing the Absorption tests of *Luffa Cylindrica* fibers (*LCFs*).

Chemical composition, surface area, size, and immersion period are among of the variables that influence *Luffa Cylindrica* fibers (*LCFs*) capacity to absorb liquids or solutions. Under normal temperature settings, Fig 4 displays the *LC* fibers’ absorbency in mineral water, seawater, vinegar, sodium bicarbonate, and ethanol. The findings demonstrate that the *Luffa Cylindrica* fibers (*LCFs*) were able to absorb up to 191%, 195%, 236%, 239%, and 134% of their dry mass, respectively, following a 24-hour immersion in mineral water, seawater, CH3COOH, NaHCO3, and C2H6O. Following a 72-hour soak in C2H6O, CH3COOH, NaHCO3, seawater, or mineral water, these fibers can absorb up to 142%, 200%, 250%, 243%, and 195% of their dry weight, respectively. The pace at which *LCFs* absorb water is high. Their strong internal arrangement and excellent hydrophilic qualities account for this capacity [49]. Due to *LCFs* saturation, the absorption result at the test’s conclusion, after 504 hours, stayed unchanged.

### 3.4. Stress-strain behavior of the fibers

**Fig 5:**
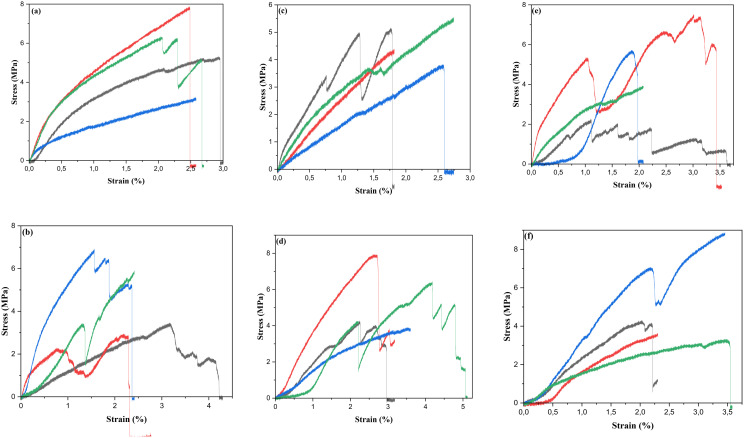
Stress-strain graphical curves for (a) dry fibers, (b) fibers immersion in mineral water, (c) fibers immersion in seawater, (d) fibers immersion in CH3COOH, (e) fibers immersion in NaHCO3, and (f) fibers immersion in C2H6O of *Luffa Cylindrica* fibers (*LCFs*).

**Fig 6:**
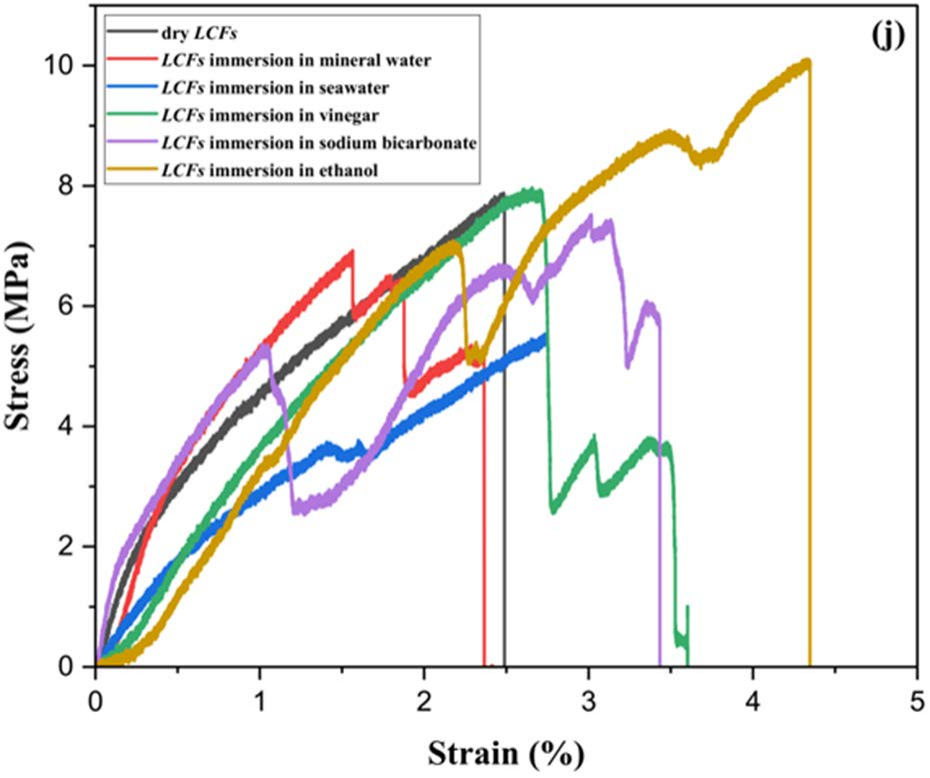
Maximum elongation comparison for untreated (dry) and treated *Luffa Cylindrica* fibers (*LCFs*).

Tensile strength, elongation at break, and Young’s modulus were the three key factors affecting the mechanical qualities [50]. Climate and soil conditions, the extraction method, chemical treatments, plant aging, fiber structure, plant type, plant growth phases, and other factors can all affect the tensile strength of natural fibers [51]. Figs 5 and 6 showed the tensile test curves for dried and treated *Luffa Cylindrica* fibers (*LCFs*). In the early stages of loading, both treated and untreated *LCFs* exhibited a linear region in the stress-strain graph, which corresponds to elastic deformation. During this phase, the fibers demonstrate a proportional relationship between stress and strain, with the fibers returning to their original shape upon unloading. For untreated *LCFs*, the yield point—the transition from elastic to plastic deformation—was observed at relatively higher stress levels, suggesting that untreated fibers maintain structural integrity before significant deformation occurs. On the other hand, fibers treated with vinegar and sodium bicarbonate exhibited a lower yield stress, implying that these treatments might reduce the fiber’s ability to resist deformation before yielding. This could be due to changes in the chemical structure of the fiber, such as the partial removal of lignin or changes in cellulose crystallinity, which affect the fiber’s rigidity and resistance to stretching. Following the yield point, a region of plastic deformation was observed for all treated and untreated fibers. This phase is characterized by the fiber undergoing permanent deformation. Some treated fibers, particularly those treated with sodium bicarbonate, showed a distinct plateau in the stress-strain curve before ultimate failure. This plateau may indicate the presence of weak points in the fiber structure, such as micro-cracks or changes in fiber alignment due to the treatment process. In contrast, untreated fibers did not show such a plateau, suggesting they maintain a more uniform structure under strain, allowing for more continuous deformation up until failure. The results showed that the untreated dry fibers had a higher tensile strength (5.59 MPa), than the treated fibers, where the tensile test was recorded for treated with CH3COOH value (5.49 MPa) compared to NaHCO3, seawater, mineral water, and C2H6O of 4.67 MPa, 4.66 MPa, 4.60 MPa, and 4.55MPa, respectively. The same trend was observed in young’s modulus, where the value of untreated fibers was greater than of treated fibers. As shown in Table 2.

**Table 2:** Tensile analysis of *Luffa Cylindrica* fibers (LCFs).

	GL (mm)	Stress (MPa)	Strain (%)	Young’s modulus (GPa)
*Dry fibers*	20	5.59	2.56	0.47
*LCFs immersion in mineral water*	20	4.60	2.42	0.31
*LCFs immersion in seawater*	20	4.66	2.21	0.33
*LCFs immersion in CH3COOH*	20	5.49	3.26	0.17
*LCFs immersion in NaHCO3*	20	4.67	2.31	0.18
*LCFs immersion in C2H6O*	20	4.55	2.67	0.16

According to other research, Luffa fibers have a tensile strength of 6.7 MPa [52], 9.4 MPa [53], and 68.1MPa [54]. According to a recent assessment, a number of factors, including the growing region, plant duration, plant type, fiber source, location, soil type, and climate condition, might cause these numbers to differ significantly [55]. In summary, we see that the fibers’ tensile qualities are not enhanced by treatment [56]. Treatments like oxidation and mercerization are typically intended for use in other applications, such as the creation of composites, where the interfacial adhesion between the fibers and the resins is crucial [57]. The purpose of these treatments is to enhance composite characteristics [11]. Therefore, the domain of application would be determined by the treatment option. Nonetheless, it is demonstrated that vinegar enhances the fibers’ tensile qualities. Table 3 compares the mechanical properties of dry *Luffa Cylindrica* fibers (*LCFs*) with luffa Cylindrica fibers in previous research.

**Table 3:** Mechanical properties of *Luffa Cylindrica* fibers (LCFs).

*Tensile strength (MPa)*	Tensile modulus (GPa)	Elongation at break (%)	Refs
*5.59*	0.47	2.56	This study
*6.7*	–	11	[67]
*9.4*	12–13	2–3	[53]
*17*	0.750	–	[22]
*17.628*	0.076	3.681	[68]
*4.875–8.029*	1.287–1.697	5.04	[69]
*7.65*	0.021	–	[70]
*20–25*	0.070	–	[71]
*20–40*	–	–	[72]
*31*	0.072	–	[21]
*202.3*	4.5	2.5 ± 0.2	[73]

In summary, the stress-strain analysis shows that different treatments significantly affect the mechanical properties of *Luffa Cylindrica* fibers. While untreated fibers generally exhibit superior tensile strength and a more uniform deformation behavior, certain treatments, especially vinegar and sodium bicarbonate, lower the yield stress and ultimate tensile strength, making the fibers more susceptible to deformation and failure. The observed changes in the stress-strain graphs highlight the importance of carefully selecting treatment methods based on the desired mechanical performance of *Luffa Cylindrica* fibers in composite applications. Future research should focus on optimizing these treatments to balance the improved surface properties with the retention of mechanical strength, making *Luffa Cylindrica* fibers a viable alternative in environmentally friendly composite materials.

### 3.5. Weibull distribution plots analysis

**Fig 7:**
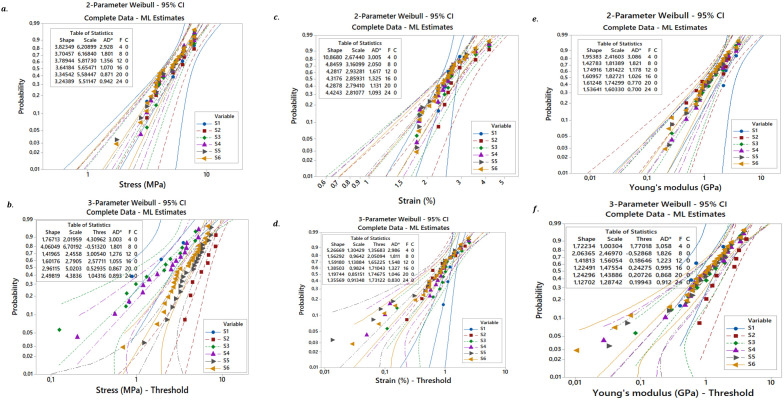
Two and three Weibull distributions for the Stress, Strain, and young’s modulus.

In Fig 7, the Weibull ML (Maximum Likelihood) statistical distribution curves are displayed with two and three parameters of the mechanical characteristics derived from the experimental results, specifically the Young’s modulus for the *Luffa Cylindrica* fibers (*LCFs*) and ultimate stress and strain for the six test batches of the studied samples (4, 8, 12, 16.20 and 24 tests).where predicting the mechanical behavior of the fibers (*LCFs*) at various test batches, which showed a wide range of findings, is one of the primary features of the two- and three-parameter distribution of the Weibull model. Fig 7 shows that the mechanical treatments of the natural *Luffa Cylindrica* plant fiber produced experimental findings that are satisfactorily described. They display quasi-linearity and quasi-overlap with a small offset between them, and they appear to fit linearly. It is evident that the two-parameter Weibull distribution provided mechanical property values that were reasonably close to the average values found through experimentation.

### 3.6. ANOVA analysis

The ANOVA results for six group’s tensile strength test are shown in Table 4; Where each group contains four samples. The number, average, and variation of the ANOVA analysis on the outcomes of the tensile strength assessment for the six groups are summarized in Table 4. The dry fibers sample has a better value than the other samples, according to the statistics. This is consistent with the experimental results shown in Table 2.

**Table 4:** ANOVA summary.

*Sample*	Tensile strength (MPa)
*S1: Dry fibers*	5.593
*S2: LCFs immersion in mineral water*	5.545
*S3: LCFs immersion in seawater*	5.251
*S4: LCFs immersion in CH3COOH*	5.089
*S5: LCFs immersion in NaHCO3*	5.003
*S6: LCFs immersion in C2H6O*	4.928

Table 5 shows the results of the ANOVA; the sum of squares within groups is 272.48 and the square sum of the inter-group ANOVA is 49.50, These two parameters, which have degrees of freedom of 5 and 78, respectively, show the correlation of variation in the population of tensile strength. Table 5’s findings indicate that there is a strong association between the parameters under investigation and the response. Syafri et al. [58] are supported by the coefficient of determination value of 0.947. Despite using natural fibers in this study, the coefficient of determination produced a respectably high result. The confidence and dependability of the data are increased when four samples are taken from each group for experimental outcomes. This finding is consistent with the coefficient of determination findings of earlier researchers (Choudhary et al. [59], and Nugroho et al. [60]).

**Table 5:** ANOVA output.

*Source of variation*	SS	df	MS	F	P-value	F crit
***Between Groups***	49.50	5	9.900	2.83	0.021	0.67
***Within Groups***	272.48	78	3.493			
***Total***	321.98	83				
***Standard Error***	0.955					
***R*** ^***2***^	0.947					

With a F value of 2.83, the ANOVA result from this study supports the hypothesis that tensile strength can be increased by ultrafine grinding and ultrasonication time. The p-value is less than the 0.021 alpha of 0.05. Therefore, in the statistical analysis of this study, the hypothesis rejected Ho. The critical F value of 0.67, which is less than the F value of 2.83, supports this parameter. With a standard error of 0.955 and a 95% confidence level, this statistical analysis is reliable. As a result, this statistical analysis’s error is within 10%.

## 4. Conclusion

The primary objective of this study was to investigate the impact of various immersion treatments: mineral water, seawater, vinegar (CH3COOH), sodium bicarbonate (NaHCO3), and ethanol (C2H6O) on the tensile properties, surface morphology, and moisture absorption behavior of *Luffa Cylindrica* fibers (*LCFs*), to assess their suitability for use in eco-friendly composite materials. The results of this study provide valuable insights into the potential of *LCFs* as an alternative reinforcement material for sustainable composites. The following results were obtained:

- Scanning electron microscope (SEM) analysis revealed that immersion treatments caused notable modifications to the fiber surface. For instance, fibers treated with vinegar exhibited smoother surfaces, potentially improving their compatibility with polymer matrices. These surface changes suggest that some treatments may enhance the interfacial bonding strength between the fiber and the matrix, a crucial factor for composite performance.
- The moisture absorption capacity of *LCFs* varied with treatment. Seawater and sodium bicarbonate treatments led to the highest moisture uptake, which may influence the fiber’s behavior in composite applications, especially in environments with high humidity.
- The results showed that the untreated dry fibers had a higher tensile strength (5.59 MPa), than the treated fibers, where the tensile test was recorded for treated with CH3COOH value (5.49 MPa) compared to NaHCO3, seawater, mineral water, and C2H6O of 4.67 MPa, 4.66 MPa, 4.60 MPa, and 4.55MPa, respectively.
- Untreated *LCFs* demonstrated the highest tensile strength (5.59 MPa), out-performing all treated fibers. While the immersion treatments led to a decrease in tensile strength, they still contributed to significant changes in the surface and moisture absorption properties of the fibers.
- The close agreement between the experimental results and the Weibull distribution is significant for several reasons. Firstly, it validates the robustness of the experimental data, providing confidence in the accuracy of the measured tensile strengths across the different treatment conditions. Secondly, this agreement suggests that the Weibull distribution is a reliable statistical tool for predicting the mechanical behavior of natural fibers, which is essential for future studies and practical applications. The ability to model and predict fiber performance using a well-established distribution like Weibull offers a valuable framework for engineers and researchers to assess the suitability of *Luffa Cylindrica* fibers in real-world composite applications, aiding in the design of more efficient and sustainable materials.

Finally, while untreated *Luffa Cylindrica* fibers (LCFs) demonstrated superior tensile strength, immersion treatments led to changes in fiber properties that may enhance other important attributes for composite applications, such as surface roughness and moisture absorption. The study found that seawater, vinegar, and sodium bicarbonate treatments induced significant changes in the fibers’ morphology and moisture behavior, which, while slightly reducing tensile strength, may improve the fibers’ overall suitability for use in eco-friendly composite materials. The combination of experimental testing and statistical modeling provided a comprehensive analysis of *LCFs’* performance, laying the groundwork for future work aimed at optimizing these fibers for industrial applications.
